# Host Responses to Live-Attenuated ASFV (HLJ/18–7GD)

**DOI:** 10.3390/v14092003

**Published:** 2022-09-10

**Authors:** Yuqin Fan, Weiye Chen, Chenggang Jiang, Xianfeng Zhang, Ying Sun, Renqiang Liu, Jingfei Wang, Decheng Yang, Dongming Zhao, Zhigao Bu, Xijun He

**Affiliations:** State Key Laboratory of Veterinary Biotechnology, Harbin Veterinary Research Institute, Chinese Academy of Agricultural Sciences, Harbin 150069, China

**Keywords:** HLJ/18-7GD strain, ASF, antibody, cytokine, T cell

## Abstract

African swine fever (ASF) is a highly contagious and fatal disease caused by the African swine fever virus. Recently, the multigene family and CD2v gene-deleted ASF vaccine candidate HLJ/18-7GD was found to be safe and effective in laboratory and clinical trials. However, the immune-protective mechanisms underlying the effects of HLJ/18-7GD remain unclear. We assessed samples from pigs immunized with a single dose of 10^6^ TCID_50_ HLJ/18-7GD. We found that pigs immunized with HLJ/18-7GD showed high levels of specific antibodies. T lymphocyte subsets (helper T cells (Th); cytotoxic T lymphocytes (CTL); double-positive T cells (DP-T cells)) were temporarily increased in peripheral blood mononuclear cells (PBMCs) after HLJ/18-7GD immunization. Once the HLJ/18-7GD-immunized pigs had been challenged with virulent HLJ/18, the percentage of Th, CTL, and DP-T cells increased significantly. PBMCs extracted from the pigs induced higher levels of CD8^+^ T cells after infection with the HLJ/18 strain in vitro. The levels of GM-CSF, IFN-γ, and TNF-α were upregulated at 7 days post-inoculation; this finding was contrary to the results obtained after HLJ/18 or HLJ/18ΔCD2v infection. The immune protection from HLJ/18-7GD resulted from many synergies, which could provide a theoretical basis for HLJ/18-7GD as a safe and effective ASF vaccine.

## 1. Introduction

African swine fever (ASF) is a devastating, highly lethal, and contagious viral disease caused by the African swine fever virus (ASFV). ASFV causes sporadic outbreaks in sub-Saharan Africa and Sardinia, Italy. A transcontinental spread of genotype II ASFV from southeast Africa to Georgia occurred in 2007. Since then, ASF has spread to the Russian Federation and eastern and western Europe, reaching Belgium in September 2018 [1]. In August 2018, the ASF epidemic in China, the world’s largest pig producer, resulted in disastrous consequences for the pork industry.

ASFV is a large, enveloped virus with a linear double-stranded DNA genome of approximately 170–190 kbp and encodes over 150 protein genes, depending on the isolates [2,3,4]. The variations in the genome length are mainly due to insertions and deletions of genes within five multigene families (MGFs) [5,6]. The genome contains up to 167 genes, including those encoding enzymes for the replication and transcription of the viral genes. MGF360 and MGF505 play a role in inhibiting the induction of interferon (IFN)-α/β [7,8]. Deletion of these genes from the virulent isolate Benin 97/1 or ASFV-G reduced virulence and produced two promising attenuated live vaccine candidates, BeninΔMGF [9] and ASFV-G-ΔMGF [10]. Further, the naturally attenuated isolates OURT88/3 [11,12,13] and ASFV/NH/P68 [14], along with the deletion of several genes of MGFs, provide a model for defining the protective anti-virus immune response. Another important ASFV virulence-associated gene is *pE402R* (also named CD2v), which encodes for an outer envelope protein [15] and is mainly responsible for the intracellular transport of viruses, inhibiting lymphocyte proliferation, and interacting with cellular AP-1 protein [16]. The deletion of CD2v or CD2-like gene altered the virulence of ASFV isolates [2,17,18]. In 2020, the Harbin Veterinary Research Institute of the Chinese Academy of Agricultural Sciences used a DNA homologous recombination technique and successfully constructed a seven-gene-deleted ASFV HLJ/18-7GD by deleting gene segments encoding seven different immune escape proteins, including MGF505-1R, MGF505-2R, MGF505-3R, MGF360-12L, MGF360-13L, MGF360-14L, and CD2v [19]. They confirmed that HLJ/18-7GD was safe and effective in laboratory and clinical trials. HLJ/18-7GD was fully attenuated in pigs, did not convert to a virulent strain, and provided complete protection against lethal ASFV challenge in specific pathogen-free (SPF) pigs and commercial pigs [19]. Therefore, HLJ/-18-7GD could be used as a prophylaxis for ASF. However, little is known about the mechanisms underlying ASFV protection, although seminal evidence has demonstrated the key role of humoral responses and specific CD8^+^ T cells in immune protection. Therefore, an improved understanding of virus–host interactions is a high priority in vaccine development.

In order to explore the host response to HLJ/-18-7GD, we immunized SPF pigs with a single dose of 10^6^ TCID_50_ HLJ/18-7GD for 60 days and collected blood and tissue samples at a specific time for immunopathology studies. This study indicated that the immune protection of HLJ/18-7GD resulted from many potentially synergic immune mechanisms. We found that pigs immunized with HLJ/18-7GD induced high levels of specific antibodies, and the T cell responses were interesting. Moreover, the kinetics of cytokines in the sera of pigs after HLJ/18-7GD immunization were in contrast to the results observed after HLJ/18 or HLJ/18ΔCD2v infection.

## 2. Materials and Methods

### 2.1. Virus Strains and Cells

The following three different ASFV strains were used in the in vivo and in vitro experiments: ASFV Pig/Heilongjiang/2018 (HLJ/18), HLJ/18ΔCD2v, and HLJ/18-7GD, which were provided by the Harbin Veterinary Research Institute (HVRI) of the Chinese Academy of Agricultural Sciences (CAAS). ASFV Pig/Heilongjiang/2018 (HLJ/18) was isolated from field samples in China as previously described [20]. HLJ/18ΔCD2v was constructed by deleting gene segments encoding CD2v, and HLJ/18-7GD was constructed by deleting gene segments encoding seven different proteins: MGF505-1R, MGF505-2R, MGF505-3R, MGF360-12L, MGF360-13L, MGF360-14L, and CD2v as previously described [19].

Pulmonary alveolar macrophages (PAMs) were derived from the lungs of SPF pigs according to the established procedures [21]. K562 cells (human chronic myeloid leukemia cells) were purchased from Solibio, China.

### 2.2. Animal Experiments

Six 6- or 7-week-old SPF pigs were intramuscularly inoculated with 1 mL of 10^6^ TCID_50_ HLJ/18-7GD, and three pigs remained uninfected and formed the control group of the study. All pigs were observed daily according to a welfare schedule to monitor their health status, and clinical signs were recorded. Blood (Vacutainer EDTA K_2_ tubes) and serum (Vacutainer serum tubes) samples were collected from all immunized pigs at different time points before and after immunization (0, 4, 7, 14, 20, 28, and 60 DPI). Tissue samples (spleen, tonsils, thymus, and intestinal, inguinal, submaxillary, bronchial, gastrohepatic, and mediastinal lymph nodes) were collected at 28 and 60 DPI.

In addition, we collected some blood samples from pigs below: (1) three SPF pigs were boosted with HLJ/18-7GD for 14 days at 28 DPI; (2) three SPF pigs were challenged with HLJ/18 for 21 days at 28 DPI; (3) three SPF pigs were challenged with HLJ/18 for 7 days; (4) three SPF pigs were infected with HLJ/18ΔCD2v for 7 days.

Experiments with 6- or 7-week-old SPF pigs from the Laboratory Animal Center of the HVRI were performed in strict accordance with the Guidelines for the Ethical Review of Laboratory Animal Welfare of China National Standard GB/T 35892-2018 [22]. The protocols were approved by the Committee on the Ethics of Animal Experiments of the HVRI of CAAS and the Animal Ethics Committee of Heilongjiang Province, China. All experiments with live ASFV were conducted within enhanced biosafety level 3 (P3+) and 4 (P4) facilities in the HVRI of the CAAS and were approved by the Ministry of Agriculture and Rural Affairs and China National Accreditation Service for Conformity Assessment.

### 2.3. Histopathology and Microscopic Tissue Evaluation

The tissue samples were fixed in 10% formalin and embedded in paraffin wax for histological analysis. For microscopic examination, sections (0.5-μm-thick sections) were stained with hematoxylin and eosin (H&E).

ASFV antigen detection was performed using specific immunohistochemical (IHC) methods, as described below. Antigen retrieval from the tissue sections was performed by microwaving the sections in citric acid sodium citrate buffer (0.1 mol/L, pH 6.0) for 23 min. Afterward, the sections were incubated for 30 min in a 3% hydrogen peroxide solution (diluted in absolute methanol) to inhibit endogenous peroxidase and blocked with 8% skimmed milk for 1 h at 37 °C for further specific antibody labeling. The mouse anti-p72 primary antibody (in-house, generously provided by the HVRI of the CAAS) was then incubated at 4 °C overnight. The secondary antibodies used included conjugated anti-mouse IgG horseradish peroxidase (1:400) (Thermo Fisher Scientific, Waltham, MA, USA), incubated at 37 °C for 1 h. Lastly, labeling was revealed with 3,3′-diaminobenzidine (Sigma-Aldrich, St. Louis, MO, USA). Positive and negative controls were included for each IHC run.

### 2.4. ASFV Detection

Real-time quantitative PCR (qPCR) was carried out on a QuantStudio 3 system (Applied Biosystems, Foster City, CA, USA) using OIE-recommended primers and probes according to the OIE-recommended procedure. In brief, ASFV genomic DNA was extracted from tissue using the TIANamp Genomic DNA Kit (Tiangen, Beijing, China), following the manufacturer’s recommendations, and used as a template to amplify a 250-bp-long fragment from ASFV p72 using 2× TaqMan PCR Mix (Beyotime, Shanghai, China). qPCR amplifications were performed in triplicate using corresponding standards (p72-pCMV, in-house) for absolute quantification.

### 2.5. Measuring Specific Antibody Responses

ASFV p72-specific antibodies in pig sera were detected using an ASFV cELISA Antibody Detection Kit (Ingenasa, Madrid, Spain), following the manufacturer’s recommendations. In brief, 50 μL sera (sample sera, positive control sera or negative control sera) were added to a 96-well microtitration coated plate per well and incubated overnight at room temperature. Then, the presence of ASFV p72-specific antibodies in sera was detected using 100 μL of specific conjugates and 100 μL of soluble 3,3′,5,5′-tetramethylbenzidine as a specific peroxidase substrate, incubated for 2 h and 30 min, respectively. After incubation, the reactions were stopped by adding 100 μL of stop solution per well. The plates were read at 450 nm wavelengths (OD_450_) using a spectrophotometer. The results were represented as the average antibody blocking rate between duplicates, which were calculated by using the following formula: [(OD_450_ of sample − OD_450_ of negative control)/(OD_450_ of positive control − OD_450_ of negative control)] × 100%.

The detection of neutralizing antibodies was adapted from a previously reported method [23]. Briefly, 200 μL 200 CCID_50_ EGFP^+^ HLJ/18 ASFV strains were mixed (1:1) with 200 μL 2-fold serial dilutions (2 to 128) of sera. After 1 h incubation at 37 °C in a 96-well plate, a 100 μL mixture was added to a new 96-well plate that was planked with 5 × 10^6^ PAMs, and cultured for 24 h at 37 °C. The EGFP^+^ HLJ/18 ASFV strains in PAMs were counted under a fluorescence microscope. Then, to follow the appearance of hemadsorption, 1% porcine erythrocytes were added to each well, and plates were observed every 12 h. As a control for the assay, sera from uninfected pigs were used.

### 2.6. Measuring Specific T-Cell Responses

Peripheral blood mononuclear cells (PBMCs) were isolated from whole blood by density-gradient centrifugation with Histopaque^®^ 1077 (Sigma-Aldrich, St. Louis, MO, USA). Isolated PBMCs were counted, resuspended in a freezing medium containing 10% dimethyl sulfoxide and 90% fetal bovine serum and stored in liquid nitrogen until use, as previously described [24].

For the flow cytometry analysis, frozen PBMCs were thawed, washed with RPMI 1640 medium (Solarbio, Beijing, China) containing 10% fetal bovine serum (Gibco, Grand Island, NY, USA), 100 IU/mL penicillin, and 0.1 mg/mL streptomycin and incubated in RPMI 1640 medium at 37 °C for 1 h in a 5% CO_2_ incubator. Trypan blue (Solarbio, Beijing, China) was used to assess cell viability. The PBMCs were then washed and resuspended in staining buffer (Thermo Fisher Scientific, Waltham, MA, USA); the cell density was then adjusted to 10^6^ cells/100 μL. The frequency of specific T cells in PBMCs was analyzed using the following antibodies: anti-pig CD4-PE (IgG2b clone 74-12-4), anti-pig CD8a-FITC (IgG2a clone 76-2-11), and anti-pig CD3ε-Alexa Fluor 647 (IgG2a clone BB23-8E6-8C8) (BD Pharmingen, San Diego, CA, USA), according to a previously reported method [13].

Alternatively, PBMCs extracted from pigs immunized with HLJ/18-7GD for 28 days were thawed and labeled with Cell Trace Far Red Cell Proliferation Kit (Invitrogen, Carlsbad, CA, USA), and 2 × 10^6^ PBMCs were stimulated with 20 μL 1.5 × 10^6^ HAD_50_ of HLJ/18 ASFV strains for 72 h in vitro. Then, cell viability was assessed by staining with the LIVE/DEAD™ Fixable Near-IR Dead Cell Stain Kit (Invitrogen, Carlsbad, CA, USA). Finally, live cells were surface-labeled with anti-pig CD8a-FITC (IgG2a clone 76-2-11) and anti-pig CD4-PE (IgG2b clone 74-12-4) antibodies (BD Pharmingen), allowing the phenotyping of the virus-specific proliferating cells.

### 2.7. Detection of NK Cell Cytotoxicity

Effector (PBMCs) and target cells (K562 cells) were used in an LDH Cytotoxicity Assay Kit (Beyotime, Shanghai, China) to detect NK cell cytotoxicity, which was performed in 96-well round-bottom microtiter plates. The PBMCs were thawed as previously described and diluted at various concentrations to yield different effector/target ratios (E/T = 100/1, 50/1, 25/1). In brief, PBMCs (100 μL, 100/50/25 × 10^5^ cells) and K562 cells (100 μL, 1 × 10^5^ cells) were added to triplicate wells at a total volume of 200 μL. The target cells (100 μL, 10^5^ cells/well) were used for spontaneous (spont. OD_562_) and total release (total OD_562_) by incubating cells with 100 μL of RPMI 1640 medium or 100 μL 1% NP-40, respectively. The plates were centrifuged at 300× *g* for 5 min and incubated for 4 h at 37 °C in a 5% CO_2_ incubator. The supernatant (120 μL) was harvested from each sample by centrifugation at 300× *g* for 5 min. Subsequently, 60 μL of the LDH working solution was added to each well, and the plate was incubated for 30 min at room temperature (25 ± 5 °C) on a plate shaker. Reactions were stopped by adding 30 μL of 1 mol/L citric acid solution per well. The plates were read at 562 nm using a spectrophotometer. The results were represented as the NK cell-specific cytolysis rate between duplicates, which was obtained using the following formula: NK cell-specific cytolysis % = [(OD_562_ of sample − spont. OD_562_)/(total OD_562_ − spont. OD_562_)] × 100%.

### 2.8. Detection of Serum Cytokine by Luminex

Serum samples were harvested in polypropylene tubes and stored at −80 °C until use. The MILLIPLEX^®^ MAP Kit (Porcine Cytokine Bead Panel 96-well Plate Assay) and Luminex^®^x200 (Merck, Darmstadt, Germany) were used to detect serum cytokines, including granulocyte-macrophage colony-stimulating factor (GM-CSF), interferon (IFN)-γ, interleukin (IL)-1α, IL-1β, IL-1RA, IL-2, IL-4, IL-6, IL-8, IL-10, IL-12, IL-18, and tumor necrosis factor (TNF)-α, according to the manufacturer’s instructions. In brief, 25 μL of serum or standards, 25 μL of serum matrix, and 25 μL of differently colored beads were added to a 96-well plate and incubated overnight at 4 °C for 16 h. Detection antibodies (50 μL) and streptavidin-phycoerythrin (SA-PE; 50 μL) were added to each well for 2 h and 30 min at room temperature, respectively. The PE fluorescence of each bead was read by Luminex^®^200, and respective standard curves between median fluorescent intensity (MFI) and concentration (ng/mL) were used to determine the concentration of each cytokine in the porcine serum samples.

### 2.9. Analysis of Relative Gene Expression by RT-qPCR

After necropsy, tissue samples were aseptically collected, treated overnight with RNA store solution (Beyotime, Shanghai, China) at 4 °C to increase the RNA stability of the tissues, and frozen at −80 °C for further RNA isolation. RNA was isolated using RNAiso Plus (TaKaRa, Dalian, China) following the manufacturer’s instructions. The integrity, quality, and quantity of the RNA obtained were checked in 1% (w/v) agarose gels using a NanoPhotometer NP80 Touch (Implen, München, Germany). RT-qPCR was used to determine the relative expression of a panel of 10 swine gene transcripts (including IL-1α, IL-1β, IL-6, TNF-α, IL-12, IL-18, IFN-γ, IL-4, IL-10, and IFN-α); the primers are listed in Table 1. RNA isolated from each tissue samples of the immunized and control animals was reverse transcribed to cDNA using the PrimeScript^TM^ RT reagent Kit with gDNA Eraser (TaKaRa). Sample cDNA was added as a template to the TB Green Premix ExTaq^TM^ II Kit (TaKaRa), following the manufacturer’s instructions, to attain a total volume of 20 μL. The final concentration of primers in the RT-qPCR reactions was 0.4 μM. Internal normalization of the gene expression analysis was performed using β-actin as a reference gene. Relative gene expression was assessed using the 2^−ΔΔCt^ method.

## 3. Results

### 3.1. Characterization of the In Vivo Pathogenesis of Attenuated HLJ/18-7GD

In this study, we aimed to characterize the pathogenesis of HLJ/18-7GD in vivo. As expected, the animals immunized with HLJ/18-7GD did not present any ASF-related signs and remained clinically normal during the entire observation period. The temperature change lines are shown in Figure S1. Histopathology and microscopic tissue evaluations correlated with these observations. ASFV detection by IHC and qPCR in tissues (including the spleen, tonsils, thymus, and intestinal, inguinal, submaxillary, bronchial, gastrohepatic, and mediastinal lymph nodes) was negative (data not shown). Similarly, all of the uninfected pigs remained normal.

### 3.2. Host Antibody Response in Animals Infected with HLJ/18-7GD

**Figure 1:**
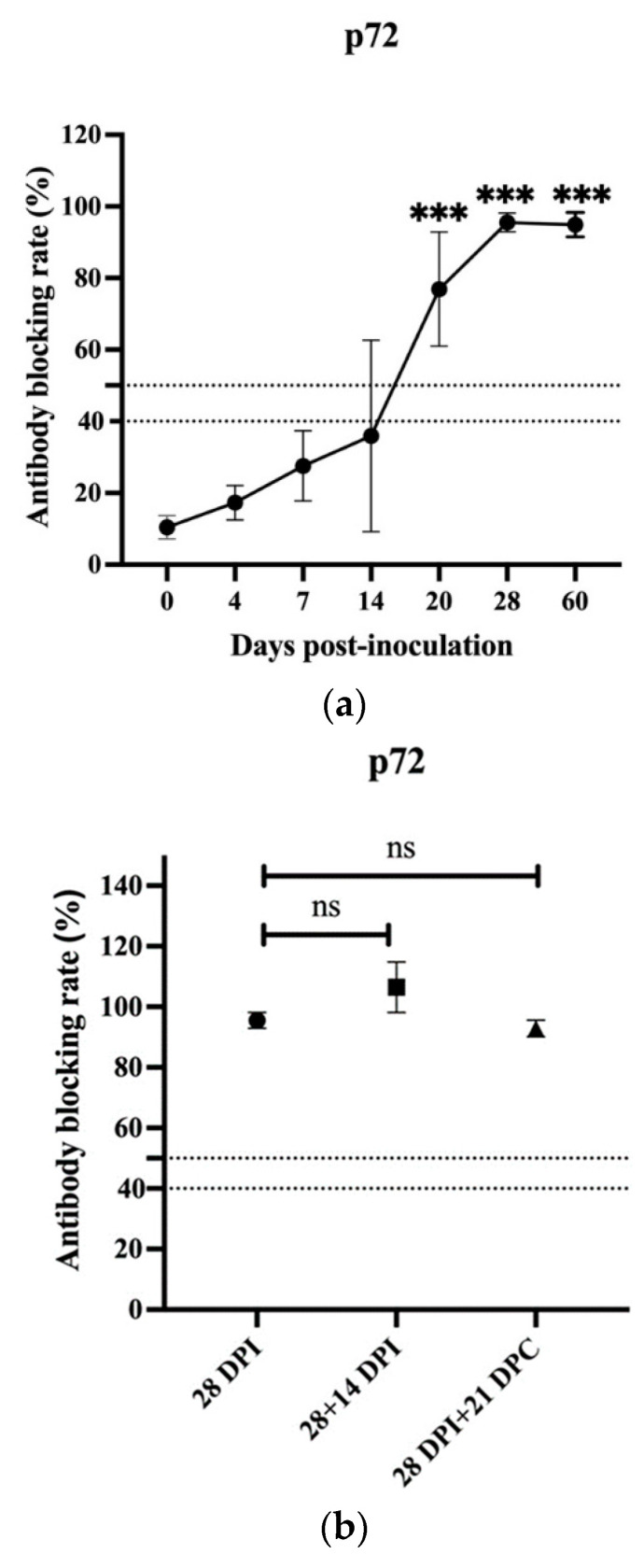
Detection of ASFV p72-specific antibodies by cELISA. (**a**) ASFV p72-specific antibodies in sera samples of pigs immunized with HLJ/18-7GD. (**b**) ASFV p72-specific antibodies in sera samples of pigs in other groups. ASFV, African swine fever virus. DPI, days post-inoculation; DPC, days post-challenge; 28 + 14 DPI, boost with HLJ/18-7GD for 14 days at 28 DPI; 28 DPI + 21 DPC, challenge with HLJ/18 for 21 days at 28 DPI. The statistical analysis was performed using a Student’s *t*-test. The asterisks indicate statistically significant differences compared with 0 DPI (**a**) or 28 DPI (**b**) (ns: no significance; *** *p* < 0.001).

To further understand the immune protective mechanisms underlying HLJ/18-7GD, the levels of p72-specific antibodies in the sera of all immunized pigs were assessed at all sampling times using cELISA. The results showed that p72-specific antibodies were first detected at 14 DPI, with only one of three animals presenting a positive antibody-blocking rate (>50%). The levels of p72-specific antibodies in all three animals increased significantly at 20, 28, and 60 DPI compared with those detected before immunization, as shown in Figure 1a. Additionally, while pigs had been boosted with HLJ/18-7GD for 14 days at 28 DPI or challenged with HLJ/18 for 28 days at 28 DPI, the levels of p72-specific antibodies levels in the pigs in these two groups also increased significantly and showed no difference compared with those detected at 28 DPI, as shown in Figure 1b. No antibodies were detected in any serum samples obtained from uninfected animals, corroborating the virological data indicating that uninfected animals were not infected by HLJ/18-7GD. Therefore, there is a close correlation between the presence of p72-specific antibodies and immune protection. The neutralization assay showed that different dilution ratios of sera from immunized pigs before or after HLJ/18 challenge did not block HLJ/18 infection of PAMs, and the hemadsorption was not inhibited (date shown in Figure S2).

### 3.3. Specific T Cell Response in Pigs Inoculated with HLJ/18-7GD

**Figure 2:**
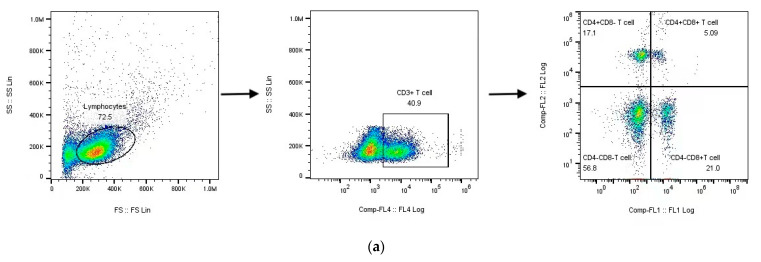

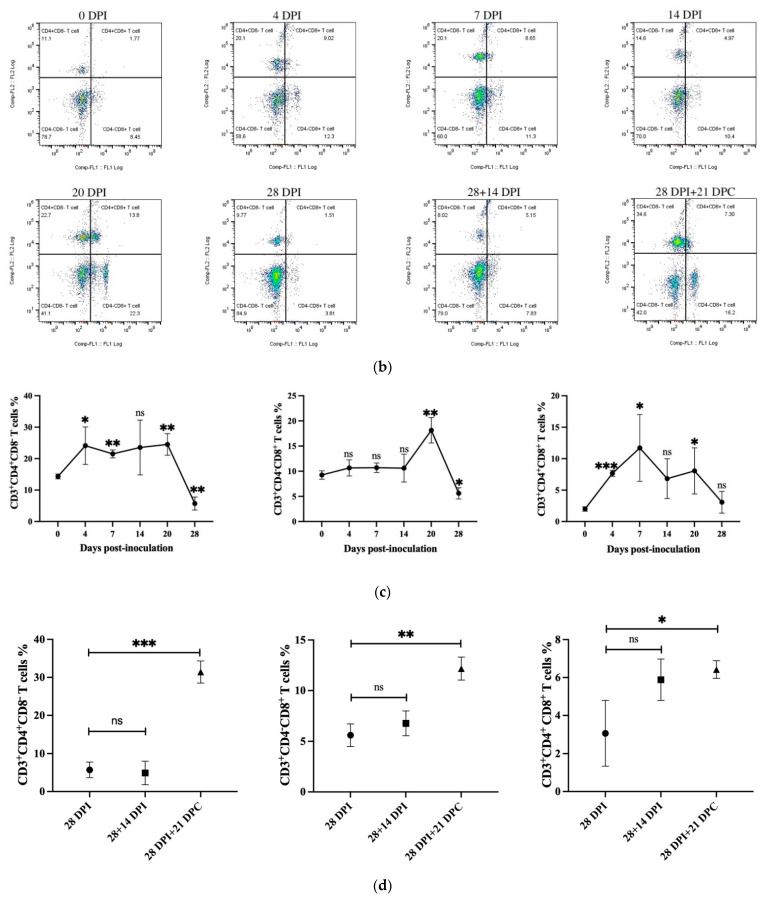
The changes in percentages of T cell subsets in PBMCs. (**a**) Gating strategy used to define T cells as CD3^+^CD4^+^CD8^−^ T cells (Th), CD3^+^CD4^−^CD8^+^ T cells (CTLs), and CD3^+^CD4^+^CD8^+^ T cells (DP-T cells) by flow cytometry analysis. (**b**) Representative data from pigs on different days after inoculation and challenge are displayed. (**c**) Mean percentages of CD3^+^CD4^+^CD8^−^ T cells, CD3^+^CD4^−^CD8^+^ T cells, and CD3^+^CD4^+^CD8^+^ T cells in PBMCs of pigs immunized with HLJ/18-7GD. (**d**) Mean percentages of CD3^+^CD4^+^CD8^−^ T cells, CD3^+^CD4^−^CD8^+^ T cells, and CD3^+^CD4^+^CD8^+^ T cells in PBMCs of pigs in other groups. DPI, days post-inoculation; DPC, days post-challenge; 28 + 14 DPI, boost with HLJ/18-7GD for 14 days at 28 DPI; 28 DPI + 21 DPC, challenge with HLJ/18 for 21 days at 28 DPI. Mean data from three pigs/time points are presented, and the error bars represent the SEM. The statistical analyses were performed using a Student’s *t*-test. The asterisks indicate statistically significant differences compared to 0 DPI (**c**) or 28 DPI (**d**) (ns: not significant; * *p* < 0.05, ** *p* < 0.01, and *** *p* < 0.001).

The importance of CD8^+^ T lymphocytes in protective immunity against ASF was previously demonstrated by depleting CD8^+^ T lymphocytes from ASF-immune pigs in vivo [12]. To test this conclusion, T cell subsets in the PBMCs of all immunized pigs were analyzed at all sampling times by flow cytometry (FCM). As shown in Figure 2a–c, the percentages of CD3^+^CD4^+^CD8^−^ cells (helper T cells (Th cells)) gradually increased after HLJ/18-7GD immunization, showing a significant increase at 4, 7, and 20 DPI compared to those detected before immunization (*p* < 0.05), followed by a significant decrease at 28 DPI (*p* < 0.01). The percentages of CD3^+^CD4^−^CD8^+^ cells (cytotoxic T lymphocytes (CTLs)) showed no significant difference within 14 days after immunization, significantly increased at 20 DPI (*p* < 0.01), and significantly decreased at 28 DPI (*p* < 0.05). The percentages of CD3^+^CD4^+^CD8^+^ cells (double-positive T cells (DP-T cells)) showed a temporary increase during the entire observation period and were significantly increased at 4, 7, and 20 DPI (*p* < 0.05), along with the recovery of pre-immunization percentages at 28 DPI.

We further analyzed the T cell subsets of the other two groups, as shown in Figure 2d. The percentages of Th cells, CTLs, and DP-T cells in pigs after two doses of inoculation showed no significant difference compared with those detected at 28 DPI. However, when the pigs were challenged with HLJ/18 for 21 days at 28 DPI, the percentages of Th cells, CTLs, and DP-T cells were significantly higher than those detected at 28 DPI (*p* < 0.05).

**Figure 3:**
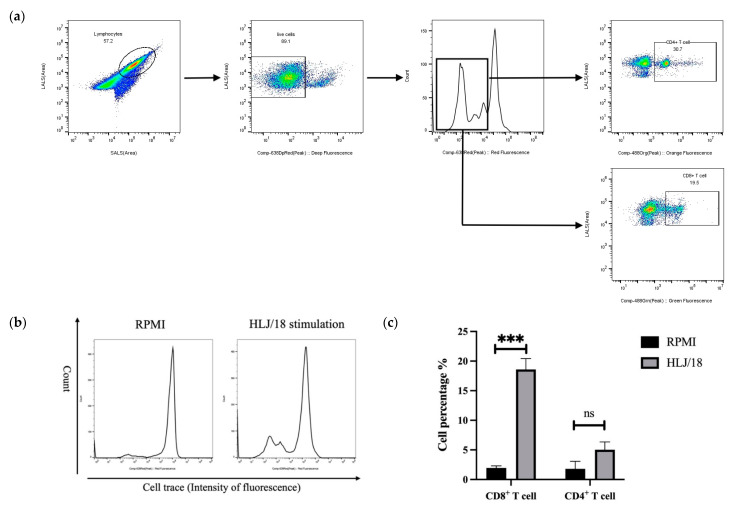
The specific cell-trace proliferation assay was performed by labeling the PBMCs obtained from pigs on day 28 post-inoculation with cell-trace and stimulated with HLJ/18 for 72 h in vitro. (**a**) Gating strategy of the proliferation assay. (**b**) Proliferation peak of PBMCs after stimulation with RPMI (control group) or HLJ/18. (**c**) The percentage of specific CD4^+^ and CD8^+^ proliferating T cells. Statistical analyses were performed using a Student’s *t*-test. Asterisks indicate statistically significant differences between experimental groups (ns: no significance, *** *p* < 0.001). PBMC, peripheral blood mononuclear cell.

Alternatively, a specific cell-trace proliferation assay was performed by labeling the PBMCs obtained from pigs at 28 DPI with cell trace and stimulating them with HLJ/18 for 72 h in vitro. The PBMCs were stained with anti-pig CD4 or anti-pig CD8 antibodies and analyzed using FCM. As shown in Figure 3, the PBMCs from pigs inoculated with HLJ/18-7GD showed specific CD8^+^ T cells capable of proliferating in vitro in response to HLJ/18 but not RPMI, and poor proliferation was observed for single-positive CD4^+^ T cells.

### 3.4. Changes in NK Cell Activity in Pigs Inoculated with HLJ/18-7GD

**Figure 4:**
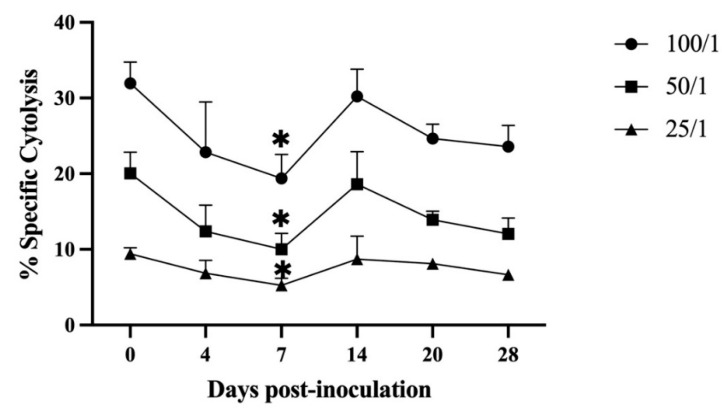
Kinetics of NK cell activity in pigs inoculated with HLJ/18-7GD at different effector/target ratios (E/T = 100/1, 50/1, 25/1). The statistical analyses were performed using a one-way ANOVA. The asterisks indicate statistically significant differences compared with 0 days post-inoculation (* *p* < 0.05).

The NK cell activity in the PBMCs of the immunized pigs was assessed at all sampling times by the LDH cytotoxicity assay. The results showed that NK cell activity initially fluctuated, decreased from 4 DPI, and reached its lowest value at 7 DPI. The NK cell activity was significantly lower than that detected before immunization (*p* < 0.05) under three different effector/target ratios (100/1, 50/1, 25/1). As the days post-immunization passed, NK cell activity began to increase, reached the pre-immunization state at 14 DPI, and remained unchanged until the end of the observation period (Figure 4).

### 3.5. Evaluation of Cytokine Levels in Serum Samples

**Figure 5:**
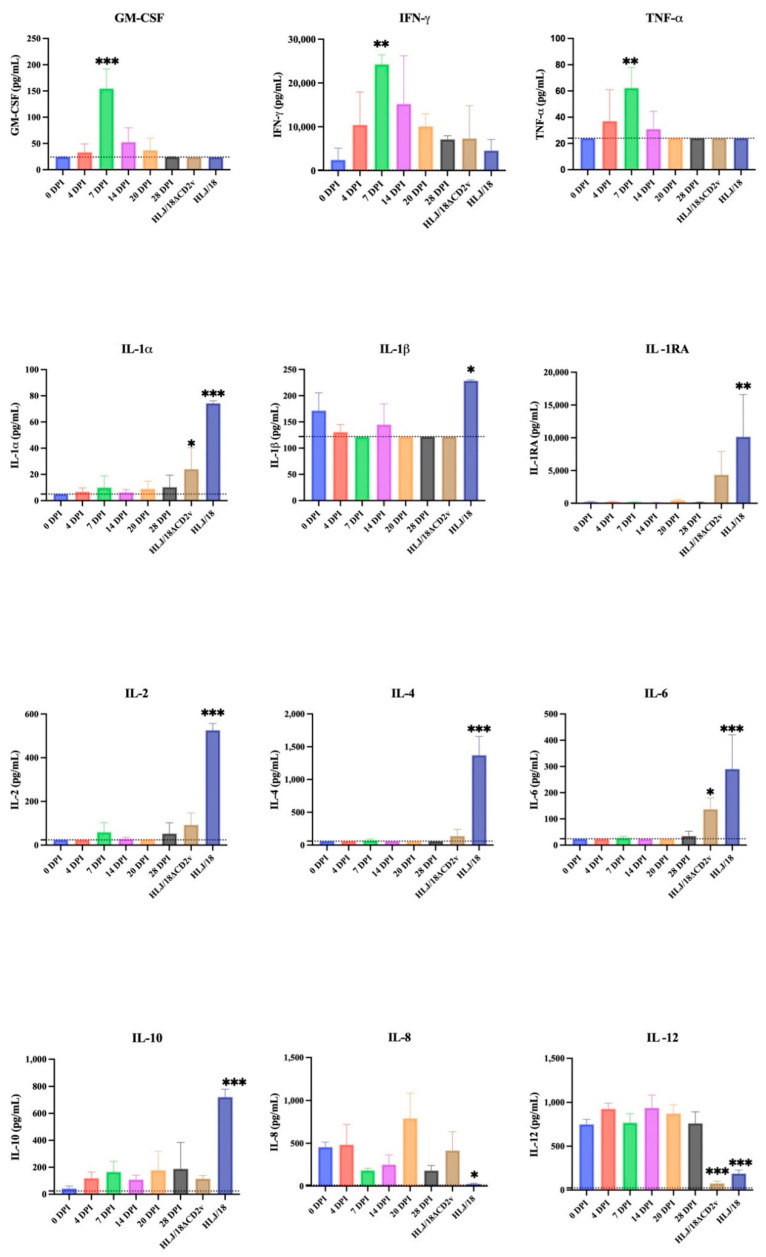
Kinetics of serum cytokines. The concentrations of GM-CSF, IFN-γ, IL-1α, IL-1β, IL-1RA, IL-2, IL-4, IL-6, IL-8, IL-10, IL-12, IL-18, and TNF-α in the sera samples of pigs immunized with HLJ/18-7GD at different time points or infected with HLJ/18 or HLJ/18ΔCD2v at 7 days post-challenge were detected via Luminex multifactor detection. Respective standard curves between median fluorescent intensity (MFI) and concentration were used to determine the concentration of each cytokine. Dotted line, minimum detection value; DPI, days post-inoculation. The statistical analyses were performed using a one-way ANOVA. The asterisks indicate statistically significant differences compared with 0 DPI (* *p* < 0.05, ** *p* < 0.01, and *** *p* < 0.001).

The levels of 13 cytokines (GM-CSF, IFN-γ, IL-1α, IL-1β, IL-1RA, IL-2, IL-4, IL-6, IL-8, IL-10, IL-12, IL-18, and TNF-α) in the serum samples of the immunized pigs were detected at all sampling times using the Luminex multifactor assay. Changes in the cytokine levels in the sera taken from pigs challenged with HLJ/18 or HLJ/18ΔCD2v for 7 days were also detected. As shown in Figure 5, the levels of pro-inflammatory cytokines IL-1α, IL-1β, IL-2, and IL-6 and anti-inflammatory cytokines IL-4, IL-10, and IL-1RA in the sera of pigs infected with HLJ/18 were significantly upregulated compared with those detected before immunization (*p* < 0.05). The levels of the pro-inflammatory factors IL-8 and IL-12 were significantly downregulated (*p* < 0.05). The changes in the levels of cytokines after HLJ/18ΔCD2v infection were similar to those observed after HLJ/18 infection. It showed that the levels of the pro-inflammatory factors IL-1α and IL-6 were significantly upregulated (*p* < 0.05); the levels of IL-2, IL-4, and IL-1RA were also slightly higher than those detected before immunization, but the differences were not significant; and the level of IL-12 was significantly downregulated compared with that detected before immunization (*p* < 0.001). After HLJ/18-7GD immunization, however, the levels of GM-CSF, IFN-γ, and TNF-α were significantly upregulated (*p* < 0.01) at 7 DPI and returned to the pre-immunization level or lower than the minimum detectable concentration, which was in contrast to the results obtained after HLJ/18 or HLJ/18ΔCD2v infection.

### 3.6. Evaluation of Relative Gene Expression in Tissue Samples

**Figure 6:**
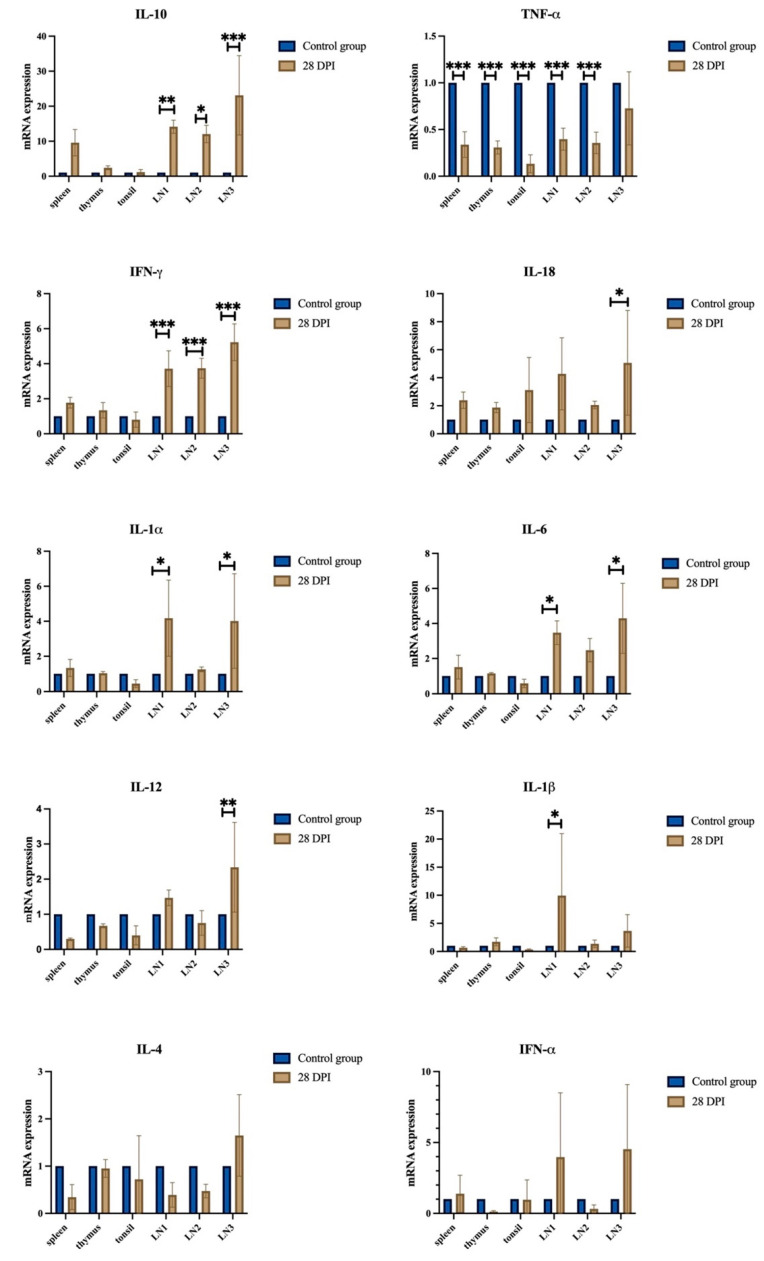
Relative gene expression analysis in tissues of HLJ/18-7GD-immunized pigs. The relative gene expression of IL-10, TNF-α, IFN-γ, IL-18, IL-1α, IL-6, IL-12, IL-1β, IL-4, and IFN-α in the sera samples of pigs immunized with HLJ/18-7GD at 28 DPI were detected via RT-qPCR. DPI, days post-inoculation; LN1, gastrohepatic lymph nodes; LN2, submaxillary lymph nodes; LN3, mediastinal lymph nodes. The statistical analyses were performed using a Student’s *t*-test. The asterisks indicate statistically significant differences compared with the control group (* *p* < 0.05, ** *p* < 0.01, and *** *p* < 0.001).

The relative gene expression of 10 cytokines (IL-1α, IL-1β, IL-6, TNF-α, IL-12, IL-18, IFN-γ, IL-4, IL-10, and IFN-α) in the tissue samples of all of the immunized pigs was detected at 28 DPI by RT-qPCR. As shown in Figure 6, many gene transcripts (e.g., IFN-γ, IL-1α, IL-1β, IL-6, IL-10, IL-12, and IL-18) were slightly upregulated in the lymph nodes compared to those detected in the control group.

## 4. Discussion

The lack of information regarding ASFV–host interactions has hindered the development of safe and effective ASF vaccines. In this study, clinicopathological, virological, and immunological parameters in pigs at different time points before and after HLJ/18-7GD immunization were evaluated to explore host response to the HLJ/18-7GD strain. We found that pigs immunized with HLJ/18-7GD could induce high levels of specific antibodies. The percentages of specific T lymphocyte subsets (Th cells, CTLs, and DP-T cells) were temporarily increased in PBMCs after HLJ/18-7GD immunization. Once the pigs immunized with HLJ/18-7GD were challenged with virulent HLJ/18, the percentages of Th cells, CTLs, and DP-T cells increased significantly. PBMCs extracted from pigs immunized with HLJ/18-7GD induced higher levels of CD8^+^ T cells after infection with HLJ/18 in vitro. HLJ/18-7GD further upregulated the levels of GM-CSF, IFN-γ, and TNF-α at 7 DPI, which was in contrast to the results obtained after HLJ/18 or HLJ/18ΔCD2v infection.

According to previous studies, B and T cell-specific responses play an important role in the protection provided by the ASFV vaccines [25]. Although early attempts to prove that specific antibodies can inhibit ASFV infection in vitro were not satisfactory [26], many studies have demonstrated that specific ASFV antibodies can reduce mortality, reduce virulence, delay the onset of infection in pigs, and provide partial immune protection [12,27,28,29,30,31,32,33]. Therefore, ASFV antibody levels can still be used as an important parameter for evaluating immune protection in the development of ASFV vaccines. We also observed that the specific p72 antibody level gradually increased after HLJ/18-7GD immunization and showed a significant difference at 20, 28, and 60 DPI compared to that detected before immunization. Additionally, the levels of p72 antibody in pigs at 28 + 14 DPI and 28 DPI + 21 DPC also increased significantly and showed no difference compared with those detected at 28 DPI. We performed a neutralization assay, and the results showed that the replication of HLJ18 strain virus was not inhibited by the sera obtained from two experimental groups. However, detection of the presence of neutralizing antibodies in ASFV has been a controversial issue for years. There is no standardized methodology for the detection of ASFV neutralizing antibodies, and the results may vary depending on the protocol used. The role of ASFV-specific antibodies in the immune protection of ASFV needs further study.

The relationship between cellular immunity and ASFV immune protection is a priority in ASFV studies. The focus of such studies includes antigen-presenting cells (such as macrophages, dendritic cells and NK cells), T cells, and regulatory T cells [25,34]. Previous studies have shown that T cells play an important role in protecting against ASFV. For example, Oura et al. found that ASFV-specific antibodies induced by the virulent strain OURT88/3 were insufficient to protect pigs against the virulent OURT88/1 challenge. CD8^+^ T lymphocytes further play an important role in the immune protection against OURT88/3 [12]. Here, we found that the percentages of Th cells, CTLs, and DP-T cells increased in a short time and showed a significant decrease or no significant difference at 28 DPI after HLJ/18-7GD immunization compared to those detected before immunization. Th cells, CTLs and DP-T cells are the main functional cells in cellular immunity and play an important role in antiviral infection. Therefore, the percentage of Th cells, CTLs, and DP-T cells increased in the early stages of immunization, indicating that the immune defense ability of the body was enhanced, which is consistent with previously described results [13]. The percentages of these cells slightly decreased or approximately returned to their pre-immunization levels at 28 DPI, indicating that the cellular immune response tended to be stable without continuous stimulation. The percentages of Th cells, CTLs, and DP-T cells in pigs after two doses of inoculation showed no significant difference compared with those detected at 28 DPI, which is likely to indicate that the level of the cellular immune response in pigs after two doses of inoculation was approximately the same as that detected after a single dose of inoculation. However, due to our limited data, the differences between two doses and a single dose of inoculation need further study. Interestingly, while the pigs immunized with HLJ/18-7GD were challenged with HLJ/18 for 21 days, the percentages of Th cells, CTLs, and DP-T cells were significantly increased compared with those detected at 28 DPI. These results indicated that Th cells, CTLs, and DP-T cells might play an important role in protecting pigs from HLJ/18 challenge, which is consistent with results from previous studies [25,35]. We further found that PBMCs extracted from pigs immunized with HLJ/18-7GD for 28 days could induce higher levels of CD8^+^ T cells after stimulation with HLJ/18 in vitro, which is also consistent with the results yielded by the attenuated strains BA71ΔCD2v and E75CV1 [18,36]. Based on these results from in vivo and in vitro experiments, we hypothesize that cellular immunity, especially CD8^+^ T cells, may play a central role in the immune protection of the HLJ/18-7GD strain.

NK cells also perform strong antiviral functions by directly killing infected cells or indirectly secreting cytokines and chemokines [37]. In 2001, Leitao et al. found that NK cell activity was enhanced at 7 DPI in animals that remained healthy after inoculation with ASFV/NH/P68 strain and developed resistance to the virulent strain L60. Conversely, animals that developed chronic lesions of ASF showed NK cell activity similar to or only slightly above that of uninfected animals [14]. This result suggests that the activation of NK cells may be an important contributing factor in preventing disease caused by non-hemadsorbing and non-virulent isolates of ASFV and in the effective induction of protective immunity against viruses [25]. In this study, NK cell activity significantly decreased at 7 DPI after HLJ/18-7GD immunization, recovered to the pre-immunization level at 14 DPI, and remained unchanged until 28 DPI. These results suggest that HLJ/18-7GD can inhibit NK cell activity at the initial stage of immunization but has little effect on NK cell activity at the later stage, which is consistent with the results yielded by the moderately virulent Malta 78 ASFV isolate [38]. Based on these results, we speculate that the minimal influence of HLJ/18-7GD on NK cell activity in pigs may be beneficial for NK cells to exert a natural killing effect on infected cells. However, since NK cell activity was not detected in the pigs infected with HLJ/18 or challenged with HLJ/18 after HLJ/18-7GD immunization, the relationship between HLJ/18-7GD and NK cell activity in pigs requires further study.

Cytokines are a variety of peptides with diverse structures that play an important role in immune regulation. They include ILs, chemokines, TNFs, and IFNs [39]. There are many previous studies showing the kinetics of cytokines induced by low-virulent ASFV isolates in vitro, such as NH/P/68 [40], BA71V [41], OURT 88/3 [42], and others. However, it is difficult to extrapolate in vitro data to what happens in vivo. In this study, we found that the levels of the pro-inflammatory factors IL-1α, IL-1β, IL-2, and IL-6 and anti-inflammatory factors IL-4, IL-10, and IL-1RA in the sera of pigs infected with HLJ/18 were significantly upregulated compared with those detected in the sera of uninfected pigs. These results are consistent with previous studies that showed that pro-inflammatory cytokines such as IL-1α, IL-1β, and IL-6 play an important role in the pathological damage caused by ASFV infection [40,43]. We also found that pigs infected with HLJ/18ΔCD2v showed a similarly significant upregulation of IL-1α and IL-6 and downregulation of IL-12 compared with the levels detected in the sera of uninfected pigs. This may have been one of the reasons why HLJ/18ΔCD2v was not completely attenuated and could not be used as an effective vaccine candidate for ASF. In contrast, pigs immunized with HLJ/18-7GD did not show an upregulation of IL-1α, IL-1β, IL-6, or other pro-inflammatory cytokines at any time points after immunization; only the levels of GM-CSF, IFN-γ, and TNF-α significantly increased at 7 DPI. GM-CSF may be involved in T cell activation. Although TNF-α is involved in regulating inflammatory processes, it can activate cellular immune responses and protect the host from a viral infection, as previously described [14,44]. Gil et al. also showed that the low virulence strain ASFV/NH/P68 could induce the expression and production of TNF-α in porcine macrophages compared to the virulent strain ASFV/L60 [45]. IFN-γ is an important factor in cellular immunity and can promote cellular immune responses by inducing the transformation of Th0 cells into Th1 cells. In conclusion, the changes in cytokines induced by HLJ/18-7GD were different following HLJ/18 infection, which may be one of the reasons why HLJ/18-7GD is completely attenuated and could be used as an effective vaccine candidate for ASF. To further explore the relationship between HLJ/18-7GD immunity and histopathological injury, we found that the relative gene expression of most cytokines (including IFN-γ, IL-1α, IL-1β, IL-6, IL-10, IL-12, and IL-18) in the lymph nodes of pigs immunized with HLJ/18-7GD for 28 days was slightly upregulated compared with the expression levels detected in uninfected pigs. H&E staining showed that the submaxillary lymph nodes obtained from pigs immunized with HLJ/18-7GD at 28 DPI showed significant reactive lymph nodule hyperplasia (Figure S3), which is usually accompanied by an inflammatory response. According to previous studies, ASFV is phagocytosed by macrophages in the tonsils, submaxillary lymph nodes, or lungs after entering the body. ASFV then binds to red blood cells (RBCs), destroys the RBC system, and causes extensive hemorrhage in lymph nodes and other tissues. An indirect cause of this may be the effect of pro-inflammatory factors [46]. Therefore, pigs immunized with HLJ/18-7GD showed that the reactive proliferation of submaxillary lymph nodes at 28 DPI might be related to the upregulation of many cytokines in lymphoid tissue.

In conclusion, this study preliminarily explored host responses to HLJ/18-7GD strain in this study. According to the above results, we hypothesize that cellular immunity, especially CD8^+^ T cell-mediated immunity, plays a central role in the immune protection underlying the HLJ/18-7GD strain, which could provide a theoretical basis for the HLJ/18-7GD strain as a safe and effective ASF vaccine candidate strain. However, our study still has some shortcomings, such as a small sample size. Further studies should be conducted to explore the immune-protective mechanisms underlying the HLJ/18-7GD strain.

## Figures and Tables

**Table 1:** List of sequences of the primers used for the RT-qPCR analysis.

Gene Name	Forward Primer (5′-3′)	Reverse Primer (5′-3′)
β-actin	CAGGTCATCACCATCGGCAACG	GACAGCACCGTGTTGGCGTAGAGGT
IL-1α	GTGCTCAAAACGAAGACGAACC	CATATTGCCATGCTTTTCCCAGAA
IL-1β	GGCCGCCAAGATATAACTGA	GGACCTCTGGGTATGGCTTTC
IL-4	TTGCTGCCCCAGAGAAC	TGTCAAGTCCGCTCAGG
IL-6	TGGCTACTGCCTTCCCTACC	CAGAGATTTTGCCGAGGATG
IL-10	CAGATGGGCGACTTGTTG	ACAGGGCAGAAATTGATGAC
IL-12	GGAGTATAAGAAGTACAGAGTGG	GATGTCCCTGATGAAGAAGC
IL-18	AGGGACATCAAGCCGTGTTT	CGGTCTGAGGTGCATTATCTGA
IFN-α	TCAGCTGCAATGCCATCTG	AGGGAGAGATTCTCCTCATTTGTG
IFN-γ	CAAAGCCATCAGTGAACTCATCA	TCTCTGGCCTTGGAACATAGTCT
TNF-α	TTATTCAGGAGGGCGAGGT	AGCAAAAGGAGGCACAGAGG

## Supplementary Materials

The following supporting information can be downloaded at: https://www.mdpi.com/article/10.3390/v14092003/s1, Figure S1: Kinetics of rectal temperatures; Figure S2: The results of the neutralization assay; Figure S3: H&E staining of the submaxillary lymph nodes.
