# Supplementary material for: Host Responses to Live-Attenuated ASFV (HLJ/18–7GD)
Source: Viruses. 2022 Sep 10;14(9):2003. doi: 10.3390/v14092003 (PMC9506386; doi:10.3390/v14092003)
Supplement: Supplementary file 1 [file viruses-14-02003-s001.zip › viruses-1828577-supplementary.pdf]

viruses

Supplementary Materials:

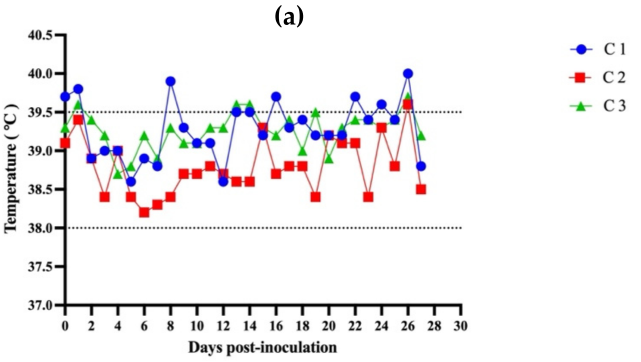

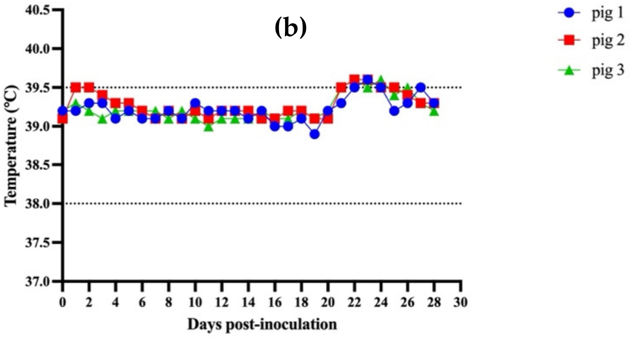

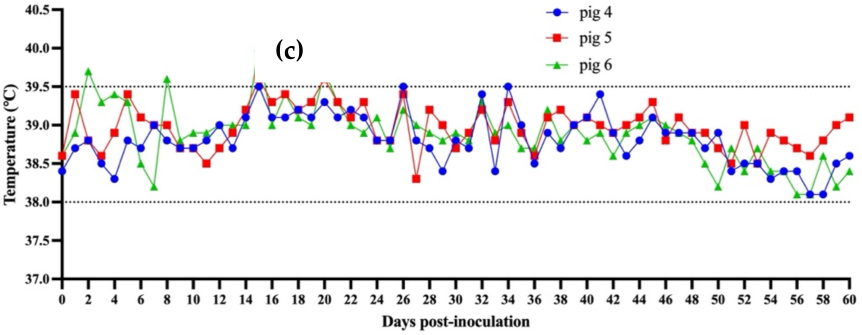

Figure S1. Kinetics of rectal temperatures. **(a)** Kinetics of rectal temperatures of uninfected pigs (pig C1/C2/C3) in the control group**. (b)** Kinetics of rectal temperatures of pigs (pig 1/2/3) immunized with 10^6 $TCID_{50}$ HLJ/18-7GD for 28 days**. (c)** Kinetics of rectal temperatures of pigs (pig 4/5/6) immunized with 10^6 $TCID_{50}$ HLJ/18-7GD for 60 days; Dotted line: The temperature range of normal pigs.

(a).

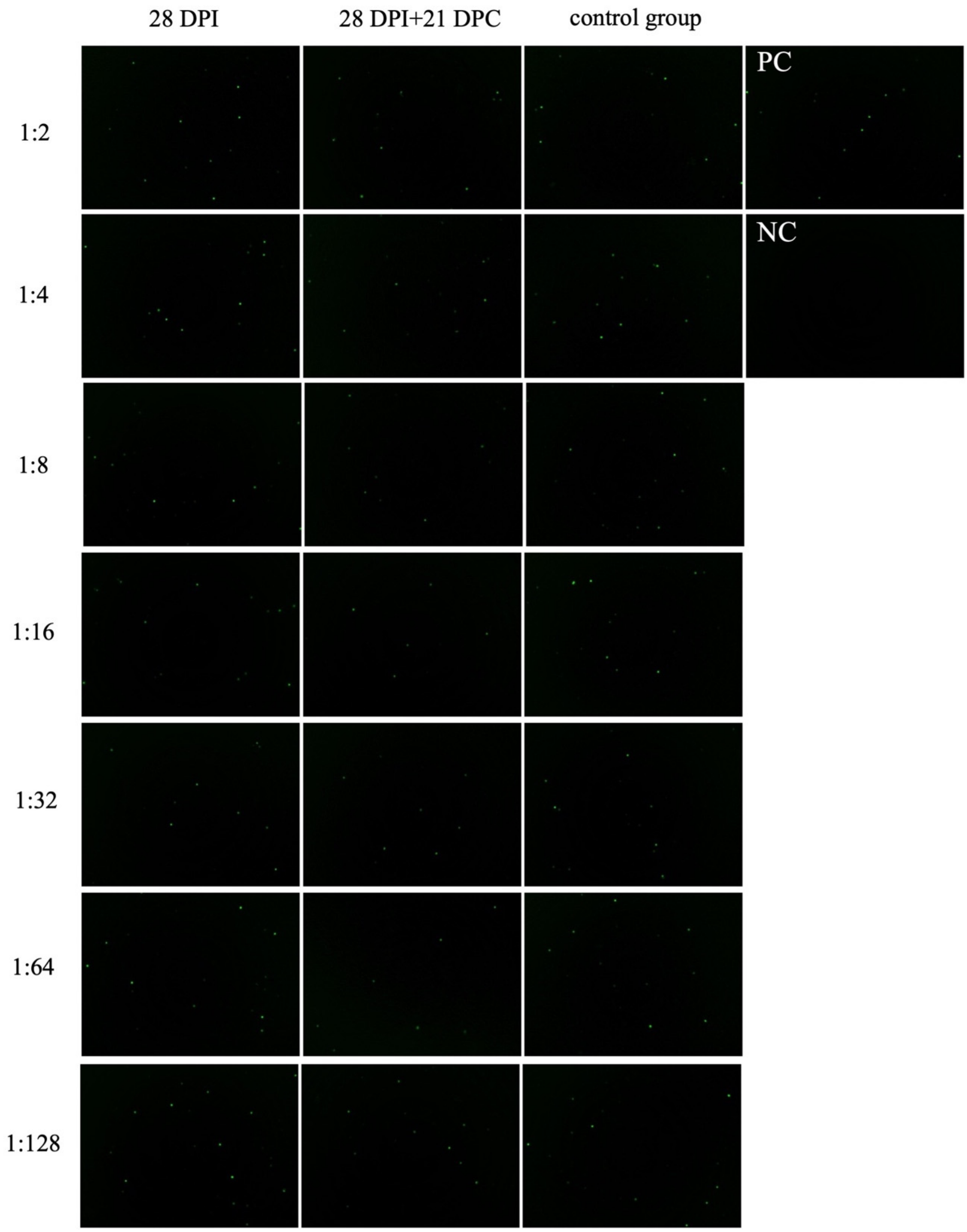
28 DPI
28 DPI+21 DPC
control group
PC
NC
1:2
1:4
1:8
1:16
1:32
1:64
1:128

(b).

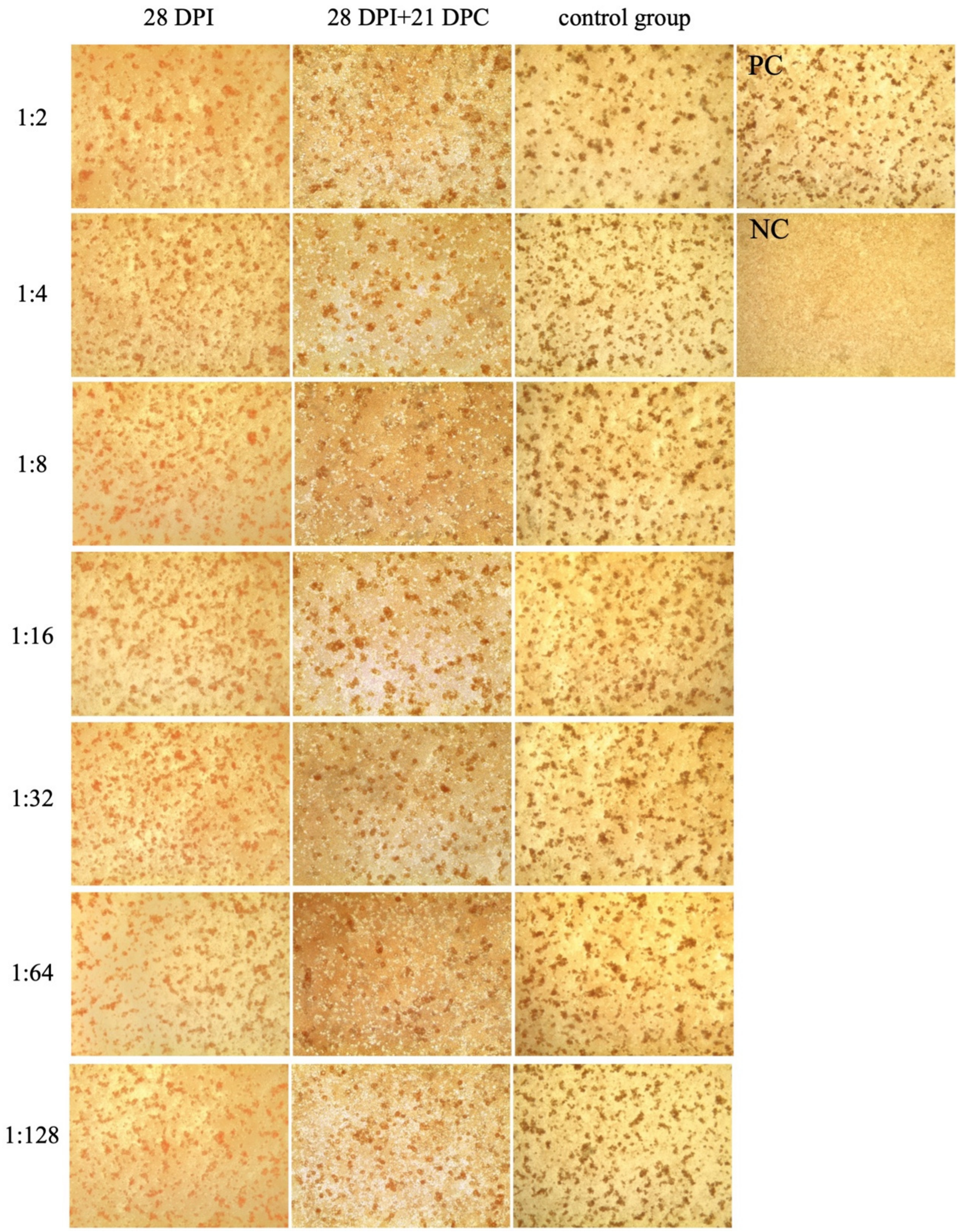

Figure S2. The results of the neutralization assay. **(a)** The EGFP⁺ HLJ/18 ASFV strain in pulmonary alveolar macrophages under a fluorescence microscope at different dilution ratios of sera (1:2 to 1:128). **(b)** The results of the hemadsorption analysis. 28 DPI were pigs immunized with HLJ/18-7GD for 28 days. 28 DPI+ 21DPC were pigs challenge with HLJ/18 for 21 days at 28 DPI. control group were uninfected pigs. PC (positive control) depicts the result without the sera. NC (negative control) depicts the result without the EGFP⁺ HLJ/18 ASFV strain. DPI, days post-inoculation; DPC, days post-chanllenge.

(a)

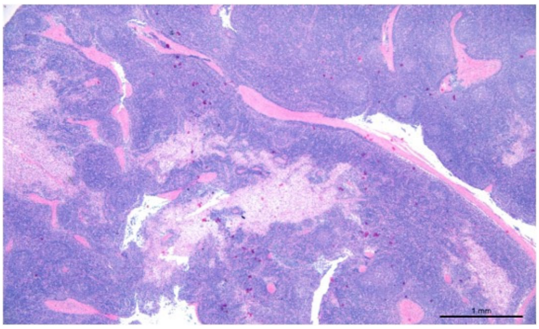

(b)

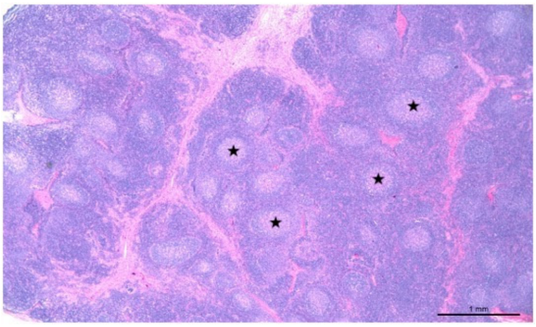

(c)

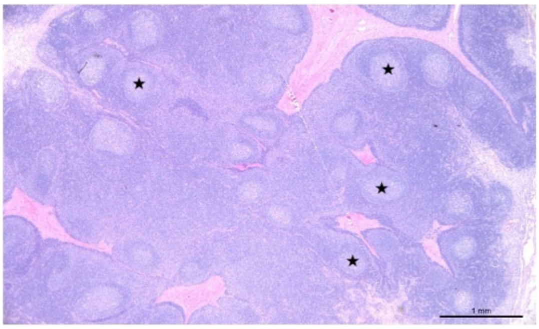

Figure S3. H&E staining of the submaxillary lymph nodes. **(a)** Pigs from the control group. **(b)** Pigs immunized with HLJ/18-7GD at 28 DPI**. (c)** Pigs immunized with HLJ/18-7GD at 60 DPI. Notable reactive lymph nodule hyperplasia was observed in the submaxillary lymph nodes of pigs immunized with HLJ/18-7GD at 28 and 60 DPI. H&E, hematoxylin and eosin; DPI, days post-inoculation.
